# Academic emotions, self-efficacy, and deep learning behaviors in online nursing education: a mediation analysis

**DOI:** 10.3389/fpubh.2026.1956924

**Published:** 2026-09-23

**Authors:** Huanhuan Xu, Xiaowen Sun, Hong Chen, Yaoyao Xia, Huihui Zhan, Fei Wang

**Affiliations:** 1 Jiangxi Institute of Technology, Nanchang, Jiangxi, China; 2 Department of Oncology, The 960th Hospital of the PLA Joint Logistics Support Force, Jinan, Shandong, China

**Keywords:** academic emotions, academic self-efficacy, deep learning, mediation analysis, nursing education, online learning

## Abstract

**Background:**

Nutrition care for older adults women with gynecologic cancers is an emerging and clinically critical competency domain in nursing education. With the accelerated shift toward online learning, understanding the psychological mechanisms that drive deep learning behaviors in this specialty area is essential for curriculum design and educational quality improvement.

**Objective:**

This study aimed to examine associations between academic emotions, academic self-efficacy, and deep learning behaviors in undergraduate nursing students participating in an online specialty nursing course, and to evaluate the mediating role of academic self-efficacy.

**Methods:**

A cross-sectional survey was conducted from September 2023 to January 2024 among third- and fourth-year undergraduate nursing students at three universities located in eastern, central, and western China. Of 540 distributed questionnaires, 487 were returned and 461 valid responses were included. The Academic Emotions Questionnaire for Online Learning (AEQ-OL), Academic Self-Efficacy Scale for Nursing Students (ASES-NS), and Deep Learning Behavior Scale (DLBS) were administered electronically. Descriptive statistics, Pearson correlation analysis, and bootstrap mediation analysis were performed.

**Results:**

A total of 461 valid questionnaires were analyzed (effective response rate 94.7%). Positive academic emotions (mean 3.72 ± 0.61) were positively associated with academic self-efficacy (*r* = 0.542, *p* < 0.001) and deep learning behaviors (*r* = 0.631, *p* < 0.001). Academic self-efficacy was also positively associated with deep learning behaviors (*r* = 0.587, *p* < 0.001). In the positive-emotion model, the indirect effect through academic self-efficacy was significant (*β* = 0.322, 95% bootstrap CI: 0.260–0.386), accounting for 50.6% of the total effect. In the negative-emotion model, the direct effect became non-significant after inclusion of academic self-efficacy, whereas the indirect effect remained significant.

**Conclusion:**

Academic emotions were significantly associated with deep learning behaviors in this online nursing education context, and academic self-efficacy showed a significant mediating association. These findings support the educational relevance of emotionally supportive learning environments and strategies that strengthen students’ confidence, while causal and temporal relationships require confirmation in longitudinal or experimental studies.

## 1 Introduction

Gynecologic cancers—including cervical, ovarian, endometrial, and vulvar cancers—create substantial care needs, particularly among older women, in whom multimorbidity, frailty, treatment toxicity, and limited nutritional reserve may complicate clinical management (1, 2). Malnutrition and treatment-related nutritional problems remain important concerns across cancer care, and nurses contribute to nutritional screening, education, monitoring, and referral. Nevertheless, undergraduate nursing students may have gaps in nutrition knowledge and counseling preparedness, supporting the need for effective educational approaches in nutrition-related oncology care (3). In the present study, geriatric gynecologic oncology nutrition care served as the specialty instructional context rather than as a separate psychometric construct.

The rapid expansion of online and blended education has increased flexibility in nursing education but has also raised concerns regarding learner engagement, sustained attention, interaction, and depth of information processing. Deep learning behaviors involve purposeful integration of new and prior knowledge, critical evaluation, transfer of knowledge to complex situations, and active inquiry. Recent studies in health-professions and nursing education indicate that learning design and psychological factors are closely related to students’ adoption of deeper learning approaches (4–6). These observations make it important to examine not only the technological environment but also the emotional and motivational processes associated with deep learning in online specialty courses.

Academic emotions are emotional experiences linked to learning activities and achievement outcomes. Contemporary applications of Control-Value Theory suggest that students’ perceptions of control and task value are systematically associated with emotions such as enjoyment, hope, anxiety, boredom, and hopelessness, which in turn are related to learning engagement and performance (7–9). In online learning environments, reduced face-to-face regulation and technology-mediated interaction may make these emotional experiences particularly salient. Evidence from online and healthcare education further suggests that positive academic emotions tend to accompany more adaptive learning experiences, whereas negative emotions may coexist with lower engagement or less favorable academic outcomes (8, 9).

Academic self-efficacy refers to students’ confidence in their ability to organize and perform learning tasks successfully. In nursing education, self-efficacy has been associated with learning engagement, self-regulated learning, academic adaptation, and readiness for professional learning (10–13). Conceptually, academic emotions and self-efficacy are closely connected: favorable emotional experiences may accompany stronger efficacy beliefs, whereas anxiety, hopelessness, or boredom may coincide with reduced confidence. Self-efficacy may therefore help explain the statistical association between academic emotions and deep learning behaviors, although reciprocal relationships are also plausible in a cross-sectional design.

The present study examined these relationships among nursing undergraduates enrolled in an online geriatric gynecologic oncology nutrition care course. The specialty content defines the educational setting, whereas the study instruments assess general academic emotions, academic self-efficacy, and deep learning behaviors. We hypothesized that: (H1) positive academic emotions would be positively associated with deep learning behaviors; (H2) academic self-efficacy would statistically mediate the association between academic emotions and deep learning behaviors; and (H3) negative academic emotions would show an inverse association with deep learning behaviors, with part of this association operating through academic self-efficacy. Because all variables were measured concurrently, these hypotheses concern associations and indirect effects rather than causal pathways.

## 2 Methods

### 2.1 Study design and setting

This cross-sectional survey study was conducted from September 2023 to January 2024 at three universities located in eastern, central, and western China, selected to provide geographic diversity in the nursing student sample. The study was reported in accordance with the STROBE checklist for observational studies. Ethics approval was reported from the Ethics Committee for Scientific Research, The 960th Hospital of the PLA Joint Logistics Support Force [Approval No. (2025) Ethics Review No. 164]. All participants provided written informed consent.

### 2.2 Participants and sampling

Third- and fourth-year undergraduate nursing students who had completed or were currently enrolled in the online specialty course were recruited using convenience sampling through participating classes at the three institutions. Eligibility criteria were: (1) full-time enrollment in Year 3 or Year 4 of an undergraduate nursing program; (2) participation in the relevant online course for at least 8 weeks; and (3) voluntary informed consent. Exclusion criteria were: (1) questionnaire missingness >20%; and (2) enrollment in a non-nursing major. G*Power 3.1 (effect size *f*^2^ = 0.15, *α* = 0.05, power = 0.90, five predictors) indicated a minimum sample of 172. To allow for nonresponse and incomplete questionnaires, 540 questionnaires were distributed. A total of 487 were returned; 26 questionnaires with >20% missing responses were excluded, leaving 461 valid questionnaires for analysis (effective response rate 94.7%).

### 2.3 Measurement instruments

#### 2.3.1 Academic emotions questionnaire for online learning (AEQ-OL)

The 40-item AEQ-OL used in this study assessed four positive emotions (enjoyment, hope, pride, relief) and four negative emotions (anxiety, hopelessness, boredom, shame) on a 5-point Likert scale. The wording was adapted to the online learning context. Recent healthcare-education research supports the psychometric usefulness of context-adapted Achievement Emotions Questionnaire formats (14). In the present sample, Cronbach’s α was 0.91 for the positive-emotion composite and 0.88 for the negative-emotion composite, and CFA supported acceptable structural fit. However, the present study did not directly test measurement invariance between online and face-to-face settings, and online-specific experiences such as technological anxiety, screen fatigue, or social isolation were not separately measured.

#### 2.3.2 Academic self-efficacy scale for nursing students (ASES-NS)

Academic self-efficacy was assessed using a 28-item nursing-student self-efficacy measure covering learning ability (10 items), clinical skills (9 items), and interpersonal communication (9 items), rated on a 5-point Likert scale. Higher scores indicate stronger perceived academic self-efficacy. Contemporary nursing education research supports multidimensional assessment of nursing learning self-efficacy (15). In the present sample, Cronbach’s α was 0.93 and CFA indicated acceptable construct validity.

#### 2.3.3 Deep learning behavior scale (DLBS)

Deep learning behaviors were assessed using a 24-item scale adapted for online nursing education, with four dimensions: critical information processing, knowledge integration and application, autonomous inquiry behavior, and collaborative learning. Items were rated on a 5-point Likert scale, with higher scores indicating greater use of deep learning behaviors. Recent nursing education studies support the relevance of deep learning constructs and case-based/online learning approaches in undergraduate nursing students (6, 16). Cronbach’s α was 0.92 and the content validity index was 0.91 in the present sample.

### 2.4 Data collection procedure

Questionnaires were distributed electronically via the Wenjuanxing (WJX) platform. A research assistant shared survey links through class communication groups, with a two-week completion window and one reminder on day 7. Respondents were informed that participation was voluntary and anonymous, and instructors were not present during completion. Questionnaires with >20% missing responses were excluded. For retained questionnaires with ≤20% item-level missingness, person-mean substitution within the corresponding scale or subscale was used only when at least 80% of that scale/subscale had been completed; this approach preserved the individual respondent’s response level while avoiding imputation across conceptually different constructs. Data cleaning, psychometric evaluation, statistical analyses, interpretation, and manuscript preparation were subsequently completed during the post-collection period before submission.

### 2.5 Statistical analysis

Data were analyzed using SPSS 26.0 (IBM Corp., Armonk, NY) and AMOS 24.0. Descriptive statistics were calculated for demographic and scale variables. Pearson correlation coefficients were used to examine bivariate associations among academic emotions, academic self-efficacy, and deep learning behaviors. Confirmatory factor analysis was used to evaluate the structural validity of the instruments, and Harman’s single-factor test was used as a diagnostic assessment of common method variance. Mediation analyses were conducted with 5,000 bootstrap resamples and 95% bias-corrected confidence intervals. Indirect effects were considered statistically significant when the bootstrap confidence interval excluded zero. Because the data were cross-sectional, mediation coefficients were interpreted as statistical indirect associations rather than evidence of temporal or causal mechanisms. Two-tailed *p* < 0.05 was considered statistically significant.

## 3 Results

### 3.1 Participant characteristics

Among the 461 valid respondents, the majority were female (89.4%), consistent with the demographic profile of Chinese nursing programs. Ages ranged from 18 to 24 years (mean 20.6 ± 1.3). All participants were in their third or fourth year of study, with third-year students accounting for 78.5%. Most participants (61.4%) reported prior exposure to geriatric care content, and 55.3% had previous online course completion experience exceeding two semesters. Detailed demographic characteristics are presented in Table 1.

**Table 1:** Demographic characteristics of participants (*N* = 461).

Variable	Category	*n*	%
Sex	Female	412	89.4
	Male	49	10.6
Age (years)	18–20	198	42.9
	21–22	201	43.6
	≥23	62	13.5
Year of study	Junior (year 3)	362	78.5
	Senior (year 4)	99	21.5
Residence	Urban	254	55.1
	Rural	207	44.9
Prior geriatric care exposure	Yes	283	61.4
	No	178	38.6
Online learning experience (semesters)	≤1	119	25.8
	2–3	255	55.3
	≥4	87	18.9
GPA (self-reported)	<2.5	43	9.3
	2.5–3.4	238	51.6
	≥3.5	180	39.1

GPA, grade point average (4-point scale). Data are presented as n and percentage (%).

### 3.2 Reliability and validity of instruments

CFA confirmed acceptable model fit for all three instruments: AEQ-OL (*χ*^2^/*df* = 2.34, RMSEA = 0.054, CFI = 0.941, TLI = 0.932), ASES-NS (*χ*^2^/*df* = 2.08, RMSEA = 0.048, CFI = 0.958, TLI = 0.946), and DLBS (*χ*^2^/*df* = 2.19, RMSEA = 0.051, CFI = 0.951, TLI = 0.939). Harman’s single-factor test revealed that the first unrotated factor accounted for 28.3% of total variance (<40% threshold), suggesting common method bias was not a significant concern. Details of psychometric properties are presented in Table 2.

**Table 2:** Psychometric properties of study instruments.

Scale/subscale	Items	Mean ± SD	Cronbach alpha	CVI	AVE	CR
Academic emotions questionnaire - online learning (AEQ-OL)						
Positive emotions composite	20	3.72 ± 0.61	0.91	0.89	0.54	0.92
Enjoyment	5	3.89 ± 0.74	0.87	—	—	—
Hope	5	3.76 ± 0.68	0.85	—	—	—
Pride	5	3.61 ± 0.72	0.83	—	—	—
Relief	5	3.62 ± 0.69	0.84	—	—	—
Negative emotions composite	20	2.41 ± 0.58	0.88	0.87	0.52	0.90
Anxiety	5	2.67 ± 0.71	0.84	—	—	—
Hopelessness	5	2.28 ± 0.63	0.82	—	—	—
Boredom	5	2.44 ± 0.69	0.86	—	—	—
Shame	5	2.25 ± 0.60	0.81	—	—	—
Academic self-efficacy scale for nursing students (ASES-NS)						
Total scale	28	3.58 ± 0.67	0.93	0.91	0.56	0.94
Learning ability self-efficacy	10	3.71 ± 0.69	0.89	—	—	—
Clinical skills self-efficacy	9	3.49 ± 0.74	0.88	—	—	—
Interpersonal communication self-efficacy	9	3.53 ± 0.72	0.87	—	—	—
Deep learning behavior scale (DLBS)						
Total scale	24	3.44 ± 0.72	0.92	0.91	0.53	0.93
Critical information processing	6	3.56 ± 0.74	0.87	—	—	—
Knowledge integration and application	6	3.48 ± 0.78	0.86	—	—	—
Autonomous inquiry behavior	6	3.37 ± 0.71	0.85	—	—	—
Collaborative learning	6	3.36 ± 0.76	0.84	—	—	—

SD, standard deviation; CVI, content validity index; AVE, average variance extracted; CR, composite reliability. AVE > 0.50 and CR > 0.70 indicate acceptable convergent validity.

### 3.3 Descriptive statistics and correlation analysis

Positive academic emotions were positively correlated with academic self-efficacy (*r* = 0.542, *p* < 0.001) and deep learning behaviors (*r* = 0.631, *p* < 0.001). Negative academic emotions were negatively correlated with academic self-efficacy (*r* = −0.418, *p* < 0.001) and deep learning behaviors (*r* = −0.503, *p* < 0.001). Academic self-efficacy was positively correlated with deep learning behaviors (*r* = 0.587, *p* < 0.001). Correlation matrix is presented in Table 3.

**Table 3:** Descriptive statistics and correlation matrix of main study variables.

Variable	Mean	SD	1	2	3	4
1. Positive academic emotions	3.72	0.61	—			
2. Negative academic emotions	2.41	0.58	−0.367***	—		
3. Academic self-efficacy	3.58	0.67	0.542***	−0.418***	—	
4. Deep learning behaviors	3.44	0.72	0.631***	−0.503***	0.587***	—

SD, standard deviation. ***p < 0.001 (two-tailed). *N* = 461.

### 3.4 Mediation analysis for positive academic emotions

A mediation model was estimated with positive academic emotions as the independent variable, academic self-efficacy as the mediator, and deep learning behaviors as the dependent variable. The direct association between positive academic emotions and deep learning behaviors remained statistically significant after inclusion of academic self-efficacy (*β* = 0.312, 95% CI: 0.241–0.383, *p* < 0.001), consistent with partial mediation. The total indirect effect through academic self-efficacy was *β* = 0.322 (95% bootstrap CI, 0.260–0.386), accounting for 50.6% of the total effect. These coefficients describe statistical mediation in cross-sectional data and should not be interpreted as establishing temporal causation (Table 4).

**Table 4:** Mediation effects: positive academic emotions → academic self-efficacy → deep learning behaviors.

Pathway	Effect (*β*)	SE	Boot 95% CI (lower)	Boot 95% CI (upper)	Proportion of total effect (%)
Total effect	0.634	0.041	0.553	0.715	—
Direct effect (c’)	0.312	0.036	0.241	0.383	49.4
Total indirect effect	0.322	0.032	0.260	0.386	50.6

AE, positive academic emotions; SE, academic self-efficacy; DLB, deep learning behaviors. Bootstrap samples = 5,000. CI = confidence interval. An indirect effect was considered statistically significant when its 95% bootstrap CI excluded zero.

### 3.5 Mediation analysis for negative academic emotions

A parallel mediation analysis examined negative academic emotions as the independent variable, academic self-efficacy as the mediator, and deep learning behaviors as the dependent variable. The direct association between negative academic emotions and deep learning behaviors was not statistically significant after inclusion of academic self-efficacy (*β* = −0.078, 95% CI: −0.162 to 0.007, *p* = 0.072), whereas the indirect effect through self-efficacy remained significant (*β* = −0.425, 95% bootstrap CI: −0.500 to −0.351). This pattern is consistent with full statistical mediation in the fitted cross-sectional model, but it does not establish causal direction (Table 5).

**Table 5:** Mediation effects: negative academic emotions → academic self-efficacy → deep learning behaviors.

Pathway	Effect (*β*)	SE	Boot 95% CI (lower)	Boot 95% CI (upper)	Significance
Total effect (c)	−0.503	0.043	−0.587	−0.419	*p* < 0.001
Direct effect (c’)	−0.078	0.043	−0.162	0.007	*p* = 0.072 (n.s.)
Indirect effect via SE (a × b)	−0.425	0.038	−0.500	−0.351	*p* < 0.001
a path (NE → SE)	−0.418	0.040	−0.497	−0.339	*p* < 0.001
b path (SE → DLB|NE)	0.396	0.042	0.313	0.479	*p* < 0.001
Full mediation confirmed?	Yes	—	—	—	95% CI excludes 0

NE, negative academic emotions; SE, academic self-efficacy; DLB, deep learning behaviors. n.s., not significant. “Full mediation” refers to the statistical pattern in this cross-sectional model and should not be interpreted as proof of a causal mechanism.

### 3.6 Factors associated with deep learning behaviors

Hierarchical multiple regression was performed to identify additional factors associated with deep learning behaviors, controlling for demographic variables. The full model (*R*^2^ = 0.512, *F* = 47.31, *p* < 0.001) is presented in Table 6.

**Table 6:** Hierarchical multiple regression: Predictors of deep learning behaviors (*N* = 461).

Variable	Model 1 *β*	Model 1 *p*	Model 2 *β*	Model 2 *p*	Model 3 *β*	Model 3 *p*
Demographic variables						
Sex (female vs. male)	0.094	0.046	0.071	0.091	0.058	0.164
Year of study (Senior vs. Junior)	0.138	0.003	0.112	0.012	0.093	0.031
Online learning experience	0.162	0.001	0.134	0.003	0.108	0.012
Prior geriatric care exposure	0.118	0.009	0.096	0.027	0.077	0.070
Emotional variables						
Positive academic emotions	—	—	0.421	< 0.001	0.312	< 0.001
Negative academic emotions	—	—	−0.287	< 0.001	−0.078	0.072
Academic self-efficacy	—	—	—	—	0.396	< 0.001
*R* ^2^	0.089		0.381		0.512	
Δ*R*^2^	—		0.292		0.131	
*F*	11.23***		47.82***		47.31***	

*β* = standardized regression coefficient. Model 1: demographic variables only. Model 2: Model 1 + academic emotions. Model 3: Model 2 + academic self-efficacy. ***p < 0.001. Year of study was coded as 0 = junior (Year 3) and 1 = senior (Year 4).

### 3.7 Conceptual and statistical models

Figure 1 presents the hypothesized mediation framework. Figure 2 displays the estimated associations among positive academic emotions, academic self-efficacy, and deep learning behaviors. Figure 3 compares deep learning behavior subscale scores between students with higher versus lower positive academic-emotion scores. All directional arrows in the conceptual figures represent hypothesized statistical relations rather than demonstrated causal effects.

**Figure 1:**
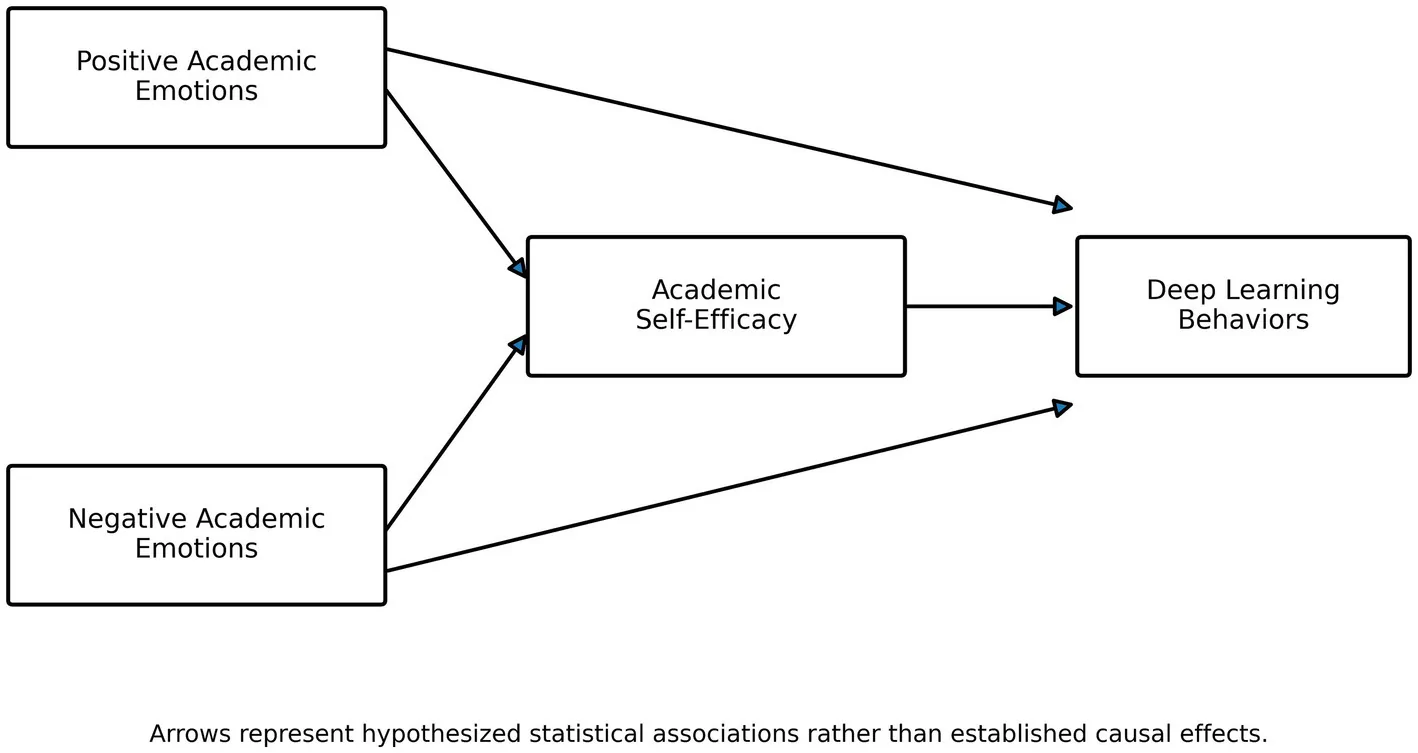
Theoretical framework depicting the hypothesized mediation model. Academic emotions (positive and negative) are modeled as statistically associated with deep learning behaviors directly and indirectly through academic self-efficacy. Arrows represent hypothesized associations rather than established causal effects.

**Figure 2:**
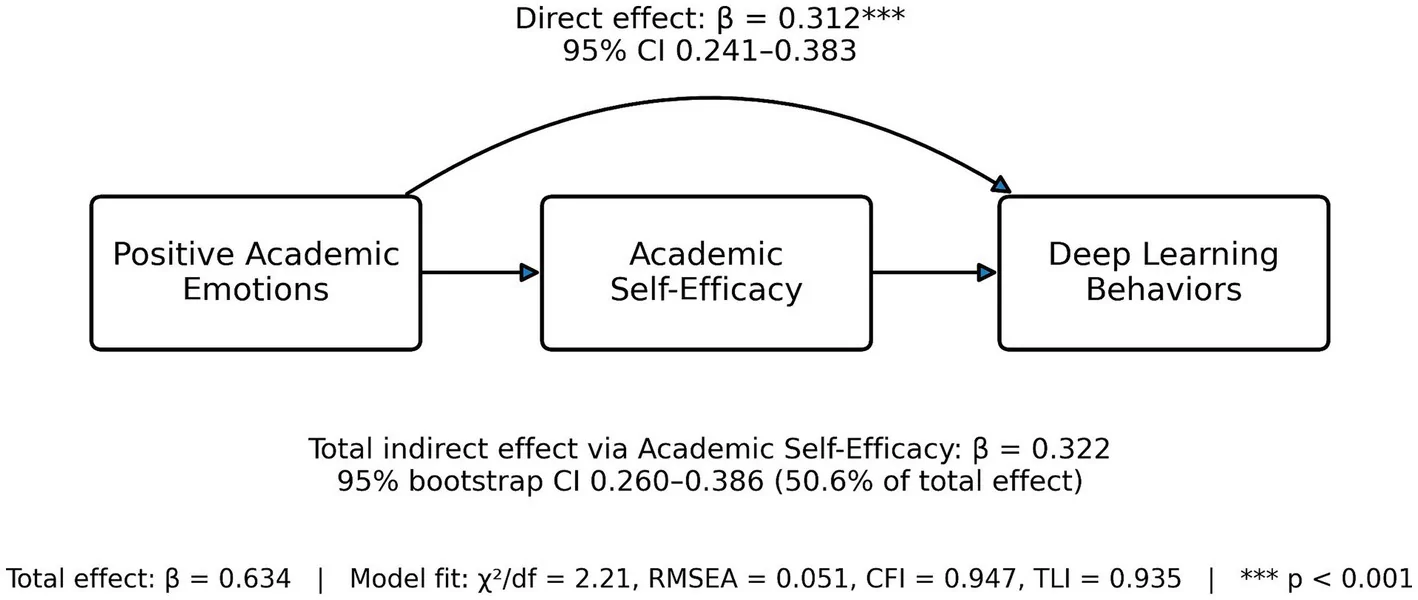
Statistical mediation model with standardized regression coefficients. Positive academic emotions are directly associated with deep learning behaviors and indirectly associated through academic self-efficacy. Model fit indices: *χ*^2^/*df* = 2.21, RMSEA = 0.051, CFI = 0.947, TLI = 0.935. ***p < 0.001.

**Figure 3:**
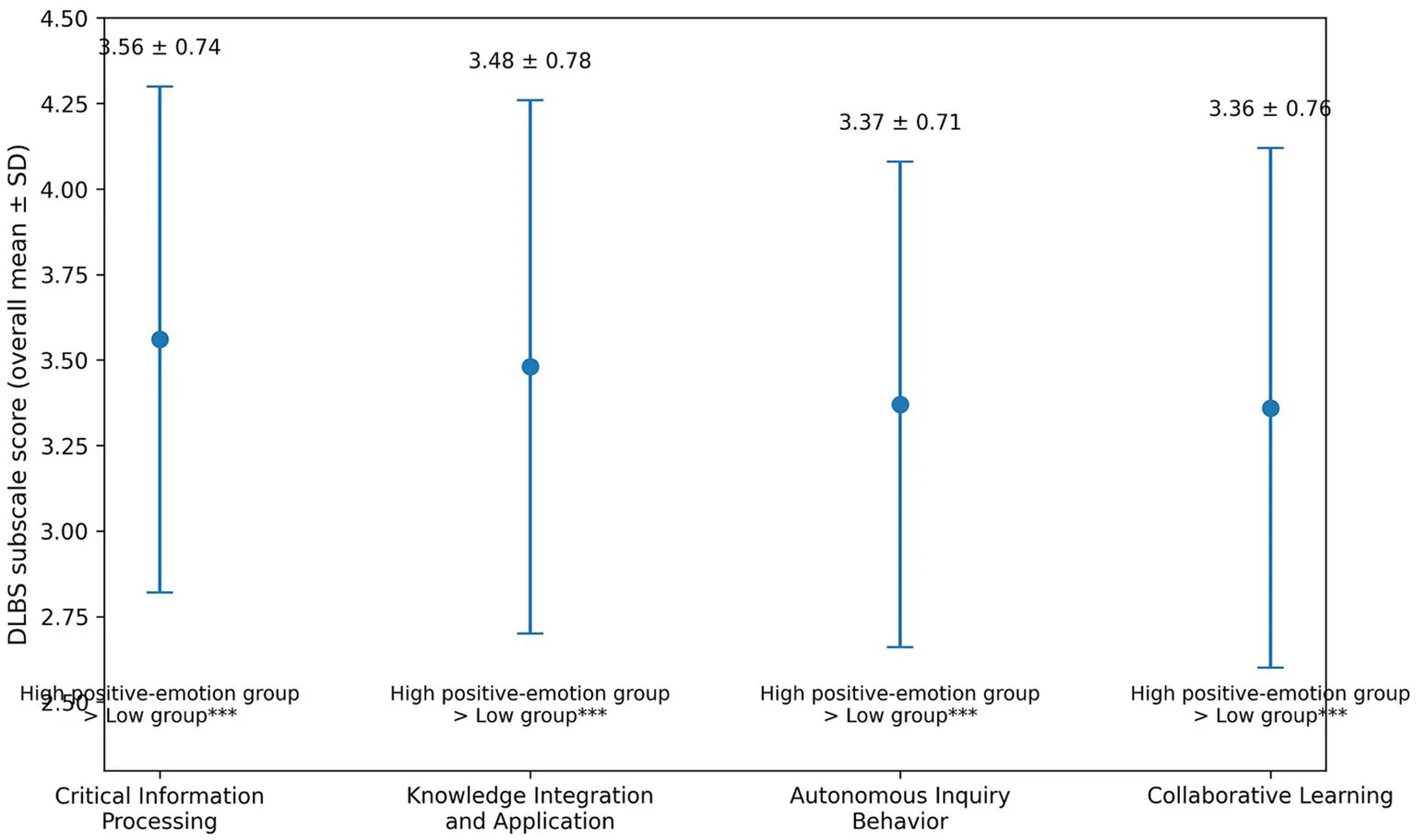
Comparison of deep learning behavior subscale scores between students with high and low positive academic emotions. Students with positive academic emotion scores at or above the median had significantly higher scores on all four Deep Learning Behavior Scale subscales than those below the median. Error bars represent ±1 SD. ***p < 0.001, independent-samples *t*-test.

## 4 Discussion

This study examined associations among academic emotions, academic self-efficacy, and deep learning behaviors in nursing undergraduates participating in an online specialty course. Positive academic emotions were positively associated with deep learning behaviors, whereas negative academic emotions showed inverse associations. Academic self-efficacy was also positively associated with deep learning and statistically mediated part of the emotion–deep-learning relationship. These findings are consistent with theoretical frameworks that link achievement emotions, efficacy beliefs, and learning engagement, while the cross-sectional design does not permit conclusions regarding temporal order or causality.

The positive association between academic emotions and deep learning behaviors is consistent with contemporary applications of Control-Value Theory and with evidence from online and health-professions education (7–9). Students reporting more positive emotions also tended to report greater critical processing, knowledge integration, inquiry, and collaborative learning. However, reciprocal explanations remain possible: students who already engage deeply with learning may experience greater enjoyment or confidence. The present findings should therefore be interpreted as evidence of covariation rather than proof that positive emotions cause deeper learning.

The negative-emotion model showed a statistically significant indirect association through academic self-efficacy, while the adjusted direct association was non-significant. This pattern suggests that self-efficacy may be an important explanatory correlate linking negative emotional experiences with deep learning behaviors. Similar relationships between psychological distress, self-efficacy, and academic outcomes have been reported in nursing students (12, 17). Nevertheless, because emotions, self-efficacy, and learning behaviors were measured simultaneously, the possibility that stronger learning behaviors reinforce efficacy beliefs—or that a third factor influences all three constructs—cannot be excluded.

The mediation findings extend simple bivariate correlations by quantifying an indirect statistical pathway through academic self-efficacy. This interpretation is compatible with recent nursing education studies in which self-efficacy has been associated with learning engagement and self-regulated learning (10, 13, 18). Importantly, the model should not be described as a causal chain. Longitudinal cross-lagged designs, repeated assessments across a semester, or experimental educational interventions would be required to establish temporal ordering and test reciprocal relationships.

The geriatric gynecologic oncology nutrition course provided a clinically relevant setting in which to examine these educational-psychological constructs. Older adults with cancer frequently experience malnutrition, sarcopenia, frailty, and treatment-related nutritional difficulties (1, 2), and nursing students require adequate preparation to recognize and respond to nutrition-related care needs (3, 19). However, the instruments used in this study assessed general academic emotions, self-efficacy, and deep learning behaviors rather than oncology-, geriatrics-, or nutrition-specific competency. Accordingly, the specialty context should not be interpreted as evidence that the observed psychological associations are unique to geriatric gynecologic oncology nutrition education.

From an educational perspective, the observed associations support testing interventions that combine emotionally supportive course design with opportunities to strengthen learning self-efficacy. Potential strategies include case-based learning, structured formative feedback, progressive mastery tasks, peer interaction, and authentic nutrition-care scenarios. Recent nursing education evidence suggests that contextualized case-based and blended nutrition education can improve aspects of deep learning and nutrition counseling capability (16, 19). These approaches should be evaluated prospectively rather than assumed to produce benefit on the basis of the present cross-sectional associations.

Several limitations should be noted. First, the cross-sectional design precludes causal inference and cannot determine whether emotions precede self-efficacy and deep learning behaviors or whether reciprocal associations exist. Second, convenience sampling from three universities and restriction to third- and fourth-year nursing students limit generalizability. Third, all principal variables were self-reported, creating potential common-method and social-desirability bias despite the psychometric checks performed. Fourth, although the AEQ-OL showed good internal consistency and acceptable CFA fit in this sample, measurement invariance between online and face-to-face settings was not tested, and online-specific experiences such as technological anxiety, screen fatigue, and social isolation were not directly represented. Fifth, geriatric gynecologic oncology nutrition care was the instructional context, but the instruments did not directly measure specialty-specific knowledge or competency. Sixth, course content and delivery differed across institutions. Finally, the interval between data collection (2023–2024) and manuscript completion reflected subsequent data cleaning, psychometric evaluation, statistical analyses, interpretation, and manuscript development; future studies should use prospectively scheduled longitudinal assessments and objective learning outcomes.

## 5 Recommendations

Nursing educators may consider incorporating emotionally supportive instructional design, timely formative feedback, mastery-oriented activities, peer interaction, and clinically authentic case-based nutrition exercises into online courses. Because the present findings are cross-sectional, these strategies should be tested in longitudinal or experimental studies using objective academic outcomes, specialty-specific competency measures, and repeated assessments of academic emotions and self-efficacy. Future research should also evaluate measurement invariance of academic-emotion instruments across online and face-to-face settings and examine online-specific emotional experiences such as technological anxiety, screen fatigue, and social isolation.

## 6 Conclusion

Academic emotions were significantly associated with deep learning behaviors among undergraduate nursing students participating in an online specialty nursing course, and academic self-efficacy showed a significant mediating association. Positive emotions were associated with higher self-efficacy and greater deep learning behaviors, whereas negative emotions showed an inverse indirect association through self-efficacy. These findings identify academic emotions and self-efficacy as potentially important targets for future educational interventions, but they do not establish causal direction. Longitudinal and experimental research incorporating specialty-specific competency and objective learning outcomes is needed before causal or practice-effect claims can be made.

## Data Availability

The raw data supporting the conclusions of this article will be made available by the authors, without undue reservation.
